# Shared regulatory sites are abundant in the human genome and shed light on genome evolution and disease pleiotropy

**DOI:** 10.1371/journal.pgen.1006673

**Published:** 2017-03-10

**Authors:** Pin Tong, Jack Monahan, James G. D. Prendergast

**Affiliations:** 1 Wellcome Trust Centre for Cell Biology and Institute of Cell Biology, School of Biological Sciences, The University of Edinburgh, Max Born Crescent, Edinburgh, Scotland, United Kingdom; 2 The European Bioinformatics Institute (EMBL-EBI), Wellcome Genome Campus, Hinxton, Cambridge, United Kingdom; 3 The Roslin Institute, The University of Edinburgh, Easter Bush, Midlothian, Scotland, United Kingdom; Buck Institute for Research on Aging, UNITED STATES

## Abstract

Large-scale gene expression datasets are providing an increasing understanding of the location of cis-eQTLs in the human genome and their role in disease. However, little is currently known regarding the extent of regulatory site-sharing between genes. This is despite it having potentially wide-ranging implications, from the determination of the way in which genetic variants may shape multiple phenotypes to the understanding of the evolution of human gene order. By first identifying the location of non-redundant cis-eQTLs, we show that regulatory site-sharing is a relatively common phenomenon in the human genome, with over 10% of non-redundant regulatory variants linked to the expression of multiple nearby genes. We show that these shared, local regulatory sites are linked to high levels of chromatin looping between the regulatory sites and their associated genes. In addition, these co-regulated gene modules are found to be strongly conserved across mammalian species, suggesting that shared regulatory sites have played an important role in shaping human gene order. The association of these shared cis-eQTLs with multiple genes means they also appear to be unusually important in understanding the genetics of human phenotypes and pleiotropy, with shared regulatory sites more often linked to multiple human phenotypes than other regulatory variants. This study shows that regulatory site-sharing is likely an underappreciated aspect of gene regulation and has important implications for the understanding of various biological phenomena, including how the two and three dimensional structures of the genome have been shaped and the potential causes of disease pleiotropy outside coding regions.

## Author summary

Where a gene’s regulatory site is disrupted by a genetic variant, its expression levels will vary between individuals depending on the version of the variant they carry. Such genetic loci, termed eQTLs, have been found to be disproportionately associated with disease and have proven to be a powerful tool for identifying the location of regulatory variation in the human genome. Despite the widespread study of eQTLs, the focus has, to date, largely been on the effect of individual variants on individual genes. The extent to which individual variants are linked to the expression of multiple nearby genes (suggestive of such variants tagging shared regulatory sites) is largely unknown. By first removing redundancy among eQTLs and then examining their effects across tissues and how they relate to how the genome folds, we show that cis-eQTL sharing is a relatively common phenomenon. We show that these master-eQTLs are not only linked to the way in which the human genome has evolved, but are also relevant to the understanding of the co-occurrence of diseases in individuals. These variants are more often linked to multiple diseases and phenotypes, which suggests that these locations are key hubs for understanding human disease risk.

## Introduction

It has been almost 30 years since the locus control region at the human β-globin cluster was identified [1], one of the first well-defined mammalian examples of a regulatory site linked to the regulation of multiple, nearby genes [2]. Subsequent studies across species have suggested that such regions may be a common feature of eukaryote gene expression [2]. However, despite their potential importance, only a handful of such regions have been identified to date. How common such master cis-regulatory sites are in the human genome remains largely unclear.

This is partly because the study of regulatory sites has focused primarily on their effect on individual genes. The study of expression quantitative trait loci (eQTLs), for example, has shown itself to be a powerful tool in the understanding of the genetic basis of gene regulation [3]. Genetic variants linked to gene expression variation (eVariants [4]) point towards the location of regulatory elements in the genome. However, traditionally, eQTL studies adopt a single variant to single gene testing approach; that is testing variants against genes one by one [3]. These analyses provide little information on the extent to which regulatory sites are shared across genes. High levels of linkage disequilibrium between variants can confound where two eVariants are tagging distinct regulatory loci, or where multiple genes are in fact linked to a single regulatory site.

Supporting the idea of substantial co-regulation in the human genome is the well characterised observation that nearby genes often display similar expression patterns across tissues [5]. A number of hypotheses have been proposed to explain this, one of which is that such genes are directly co-regulated. It has also been proposed that the similarities in expression profiles of nearby genes are simply though an artefact of their shared chromatin environment [6,7]. The latter is supported by observations that co-expressed genes are more likely to move apart over evolutionary time [6,8], suggesting that co-expression is an unwanted artefact of proximity and that selection acts to reduce the interference between the regulation of nearby genes. This apparent contradiction between studies is potentially due to the inability to distinguish between artefactual co-expression and directed co-regulation when only the expression levels of genes are analysed.

Where co-regulation does exist, the mechanism by which master regulatory sites may co-regulate multiple genes is also poorly understood. A number of models have been proposed to explain how a single site could regulate multiple genes [2,9]. These include: that transcription factor binding at the locus leads to remodelling of the chromatin across the entire locus making it more conducive for transcription; that transcription factors bind to the regulatory site which then track along to the various genes; or the promoters of the genes interact with the regulatory site via chromatin looping.

Finally, the importance of regulatory site sharing in pleiotropy, the phenomenon by which a single gene or variant is associated with more than one phenotype [10], has been largely unexplored. Traditionally pleiotropy has been thought of in terms of a particular gene being linked to multiple traits [10] but there is growing evidence that this view may be outdated. The fact that 88% of variants linked to a disease map outside transcribed regions [11] suggests that pleiotropy may also be driven by non-coding regions. Genetic variants at shared regulatory sites affect the expression levels of multiple genes and, as a consequence, multiple downstream phenotypes.

In summary, despite their potential importance to a variety of key biological processes, from understanding disease risk, to pleiotropy and the forces shaping genome evolution, little is currently known about the locations and extent of master regulatory sites in the human genome. To begin to address this we first identified shared, non-redundant, cis-regulatory sites in the human genome, which we term master-eQTLs. We then investigated the links between these sites and disease, their potential mechanisms of action and how they appear to have shaped the evolution of our genome.

## Results

### Identifying reproducible non-redundant regulatory sites in the human genome

To determine the location of master-eQTLs, we first identified the location of cis-eVariants in the human genome using a dataset of 379 European derived lymphoblastoid cell lines [12]. Genetic variants within 500kb of each TSS in the genome were tested for an association with the gene’s expression using linear regression, while controlling for any sex and population of origin effects. Robust permutation-adjusted P values were subsequently calculated for all eVariants reaching a nominal significance of p < 0.00001. A set of 441,723 cis-eVariants identified at a false discovery rate of 0.05. A final pruning step based on forward regression (see methods) was used to reduce the redundancy among the set of eVariants for each gene, and identify a final set of 5,254 conditionally independent regulatory cis-eQTLs. This pruned, non-redundant set of eVariants is provided as S1 Table.

**Fig 1:**
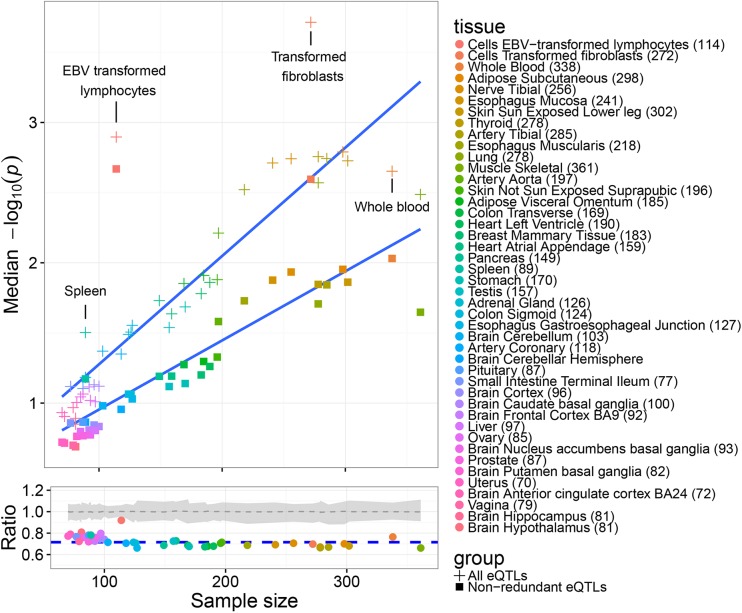
Assessing the reproducibility of eVariants across tissues. Median log transformed p values in each tissue of the GTEx dataset for those eVariants significant (q < = 0.05) in the GEUVADIS dataset before and after removing redundant variants (top panel). Sample numbers are shown in brackets after the tissue name in the legend. The corresponding points in the lower panel show the ratio of these average–log_10_(p) values for the pruned and unpruned sets (median–log_10_(p) of pruned set/median -log_10_(p) of unpruned set). The blue dotted line indicates the median of these ratios and the grey area and grey dotted line represents the range and median of the ratios observed when the independent eQTL variants were replaced with the same number of eVariants randomly selected from the background set of eVariants 1000 times. The comparatively high ratio associated with lymphoblastoid cells compared to other tissues is broadly consistent with pruning reducing spurious cross-tissue replication (S1 Fig).

To assess the quality of this set of cis-eQTLs and the impact of pruning, we explored the replication of these eQTLs across tissues using independent data from the GTEx consortium [13]. For each eVariant defined in this study, we obtained its corresponding GTEx p value in 44 tissues, including matching independent lymphoblastoid cell lines. As illustrated in Fig 1, the cis-eVariants identified in this study displayed a high level of reproducibility in related tissues such as spleen, the primary storage area of lymphocytes. In particular the conditionally independent set of cis-eVariants, each expected to correspond to a separate eQTL, showed the highest reproducibility among the corresponding dataset of lymphoblastoid cell lines, illustrating that these independent eVariants comprise a high quality collection of regulatory variants that replicate across analysis approaches and datasets. In comparison to other tissues, pruning had a comparatively modest impact on replication in the matching set of GTEx lymphoblastoid cell lines (Fig 1, bottom panel). This is broadly consistent with pruning having reduced spurious cross-tissue replication, while not substantially affecting replication across the same tissue type (see S1 Fig for further details). This pruned set of non-redundant eQTLs was therefore used in all downstream analyses.

### Master-eQTLs are common in the human genome

**Fig 2:**
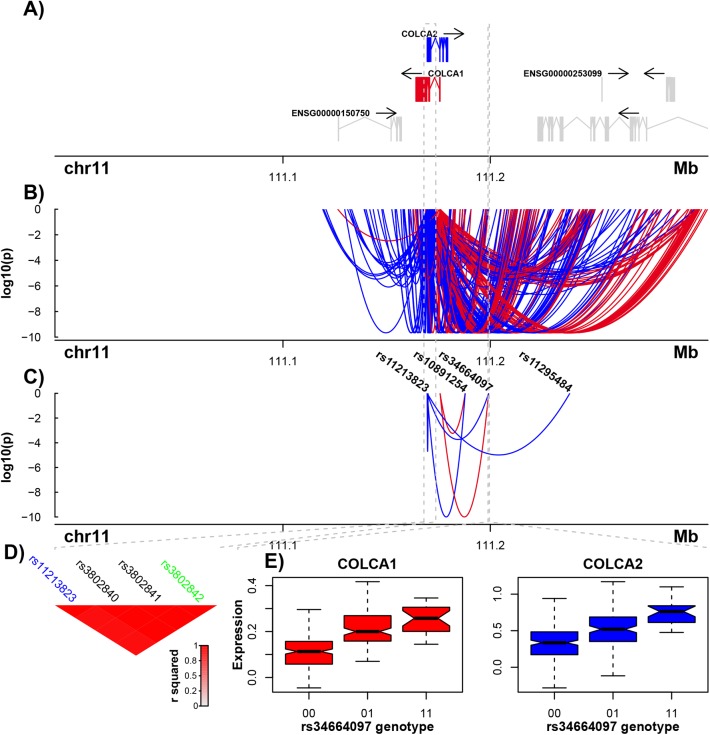
Defining the master eQTLs at the COLCA locus on chromosome 11. (A) COLCA1 and COLCA2 are orientated on opposite strands. (B) All eVariants linked to COLCA1 and COLCA2. Each eVariant is represented by a line linking the variant’s location to the TSS of the gene with which it is associated. The associated gene is also indicated by the colour of the line, and its height corresponds to its significance. (C) eQTLs remaining after removing redundancy. Two eQTLs are linked to both genes. (D) The rs11213823 variant linked to COLCA2 expression is tagging the same regulatory site as the colorectal cancer risk variant rs3802842 [28]. (E) Coregulation of COLCA1 and COLCA2 by a shared regulatory variant tagged by rs34664097.

After having identified this set of non-redundant cis-eQTLs, we assessed the extent of eQTL sharing, that is where the expression of multiple genes is linked to a single eVariant. In total 534 eQTLs, 11.8% of the total, were found to be linked to the expression of two or more genes. Fig 2 shows examples of putative shared regulatory sites at the COLCA1 and COLCA2 locus on chromosome 11. Of 4 eQTL variants independently associated with these genes, two are shared by both, consistent with previous studies demonstrating co-regulation between these genes [14].

**Fig 3:**
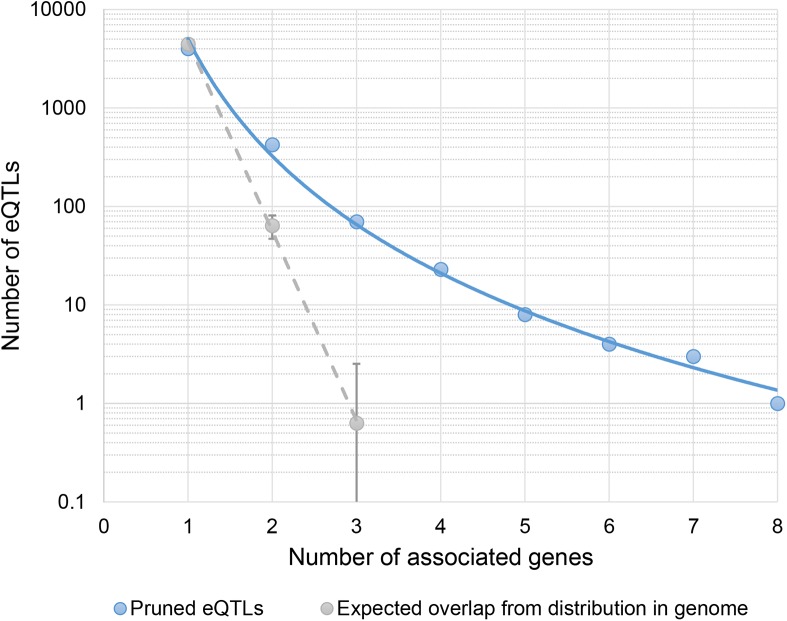
Number of master-eQTLs in the genome. The number of independent eQTLs linked to different numbers of genes (blue solid line). For comparison the number of eQTLs expected to be linked to different numbers of genes based simply on their allele frequency and distance to nearby genes is shown in grey (dashed line with 95% confidence intervals).

S2 Fig shows that the probability of a variant being a non-redundant eVariant is related to its allele frequency and distance from the corresponding gene. Independent eVariants are generally of a higher minor allele frequency and in close proximity to the gene they are linked to. This suggests regions of high gene density may show a high degree of eQTL overlap by chance. To provide a baseline frequency of eQTL sharing, we used these frequencies to determine the probabilities of eQTLs being linked to multiple genes based solely on the allele frequencies and distance to nearby genes of eVariants (see methods). Comparatively few eQTLs are expected to overlap by chance based solely on these frequencies, suggesting a bias towards shared regulatory sites in the genome (Fig 3).

### Master-eQTLs and coregulation across tissues

**Fig 4:**
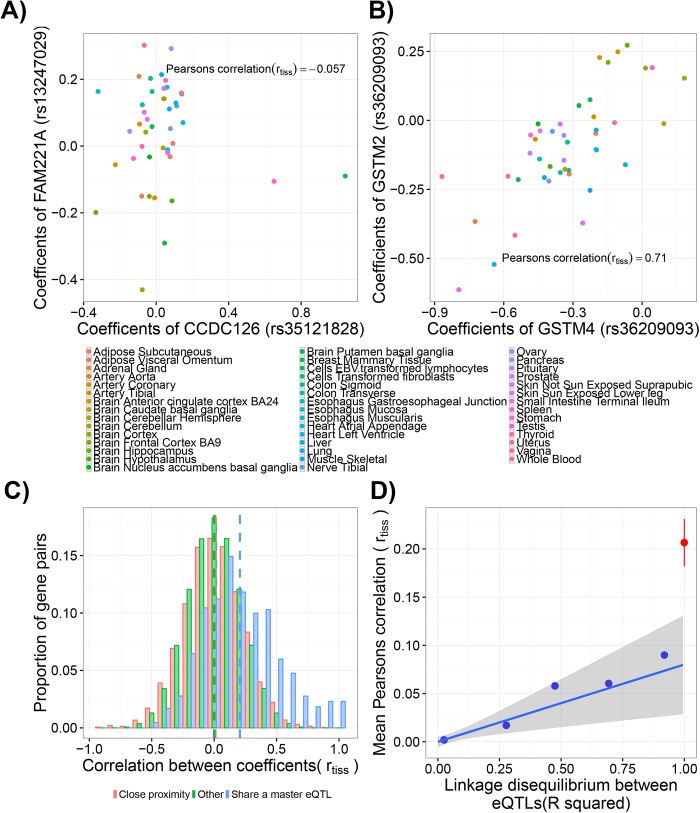
Coregulation of genes across tissues. (A) The correlation observed between the sets of tissue-specific coefficients associated with two distinct eQTL variants, rs35121828 and rs13247029, in high LD (R^2^ = 0.88). Variant ids are indicated in brackets after the name of the gene they are associated with. Each point represents the eQTL’s coefficient in that tissue, i.e. its size and direction of effect on the corresponding gene’s expression level in the associated cell type. Despite the eQTLs being in high LD, the correlation between their sets of coefficients (r_tiss_) is low. (B) The high correlation between the sets of tissue coefficients associated with a master-eQTL tagged by rs36209093. (C) The distributions of the correlations (r_tiss_) between pairwise sets of coefficients for gene-eQTL pairs where the eQTL is the same but the genes differ (share a master eQTL); where the eQTLs and genes both differ but the gene pairs were sampled 100 times to match the distribution of inter-gene distances observed between genes sharing a master-eQTL (close proximity); and all pairs of sets of coefficients where the eQTLs and genes both differ (other). Means of each group are indicated by vertical dashed lines. The master-eQTL distribution is significantly different from both other groups (Kolmogorov-Smirnov test p < 2.2x10^-16^) (D) The mean correlations (mean r_tiss_) associated with pairs of eQTLs linked by different levels of LD. The blue circles represent the mean correlations and mean LD of eQTL pairs broken down into five bins (LD between 0 and 0.2, 0.2 and 0.4, 0.4 and 0.6, 0.6 and 0.8, 0.8 and 1). The blue line and grey areas represents a fitted linear regression line through all the (unbinned) data and its 95% confidence interval respectively (R^2^ = 0.001; p = 0.0033). The red point and lines correspond to the mean correlation between sets of coefficients associated with master-eQTLs and the associated 95% confidence interval.

Although these master-eQTLs may indeed correspond to shared regulatory sites, an alternative explanation is that they are in fact two independent eQTLs in high LD. The tight genetic correlation of many eVariants means that their effects cannot be easily teased apart. However, two independent regulatory variants, affecting both distinct regulatory sites and downstream genes, will not necessarily show correlated effects on the expression of the genes across different tissues. Transcription factor binding, histone modifications and other chromatin factors at these distinct sites can change independently of one another and the underlying genotypes at these variants, leading to divergence in the expression levels of the genes across tissues. In contrast changes in transcription factor binding across tissues at shared regulatory sites are shared by each co-regulated gene, suggesting the effect of variants at such sites will have more correlated effects on associated genes across tissues than two independent, but linked, variants. This theory is illustrated in Fig 4A and 4B. Although the two eVariants, rs13247029 and rs35121828, are in high LD (R^2^ = 0.88) the estimated size and direction of their effects on the genes they regulate are not correlated across tissues. The expression levels of the genes change independently across cell types, and show different associations with the distinct eVariants. In contrast the size and direction of effect of the master-eQTL eVariant rs36209093 on the two genes it is linked to, are highly correlated across tissues. We tested to see if this is a general phenomenon and a feature of the master-eQTLs defined here, that is whether multi-eQTLs show a greater correlation in their size and direction of effect on their linked genes across tissues than would be expected if multi-eQTLs are in fact tagging two independent regulatory sites.

To test this, we obtained from the independent GTEx dataset the coefficients across 44 tissues for each of our independent eVariants. Each eQTL-gene pair’s set of 44 tissue coefficients represents the size and direction of effect of the eQTL across tissues. The Pearson’s correlation was then calculated between the sets of coefficients associated with each pair of eQTL-gene sets in the genome (r_tiss_). A high r_tiss_ indicates that the size and direction of effect of the eQTL or eQTLs on their associated genes is highly correlated.

r_tiss_ is generally low for randomly selected pairs of eQTLs (Fig 4C), i.e. the size and direction of effect of distinct eQTLs on different genes across tissues are generally uncorrelated. A similar pattern is observed if nearby gene-pairs are randomly selected to approximately match the distribution of inter-gene distances observed between genes sharing a master-eQTL. The coefficients associated with these gene pairs and their distinct eQTLs also show little correlation (low r_tiss_), suggesting two genes simply being in close proximity does not necessarily increase their level of co-regulation across tissues. However, r_tiss_ associated with master-eQTLs and associated genes are unusually high. The sets of coefficients associated with master-eQTLs and each co-regulated gene were generally more highly correlated than expected.

As the genotypes of genetically linked variants are correlated, this may partly explain correlations between associated sets of eQTL coefficients. As shown in Fig 4D, there is indeed a weak relationship between the correlations observed between the sets of coefficients associated with pairs of eQTLs (r_tiss_) and increasing levels of LD between the variants. The higher the LD between two eQTLs the greater the correlation between their size and direction of effect on the expression of their associated genes across tissues. However, extrapolating out this relationship in Fig 4D, master-eQTLs display correlations between their associated sets of coefficient (r_tiss_) that are substantially higher than would be expected from two independent master-eQTLs in perfect LD. These data suggest that master-eQTLs are not simply the result of independent regulatory variants in perfect LD, or because the genes simply share the same broader chromatin environment, but rather are likely enriched with true shared regulatory variants.

### Linking of master-eQTLs and co-regulated gene clusters via chromatin looping

To investigate the potential mechanisms by which multiple, often distant, genes may potentially be co-regulated, we tested for evidence of chromatin looping between master-eQTLs and their associated genes [15]. Two complementary Hi-C chromosome conformation datasets were used. One traditional genome-wide Hi-C analysis [16] characterising chromatin interactions genome-wide, and one higher resolution but targeted study of loops specifically associated with gene promoters [17]. In total 175 of the 1039 gene pairs sharing an eQTL (16.7%) displayed evidence of both genes looping towards the shared regulatory site (S2 Table). A high degree of overlap was observed between both chromatin conformation datasets (S3 Fig).

To assess the significance of this result we adopted a circular permutation approach [18]. This involved maintaining the size, order and interactions between Hi-C target and anchor regions, but shifting the locations of all the Hi-C regions the same random distance along a circularised version of the genome, to test how often looping between genes and regulatory regions is expected by chance (see methods for more details). Although genes that are closer together are expected to be more often linked by chromatin interactions, this circular permutation approach ensures that factors such as the distance between genes are controlled for.

**Fig 5:**
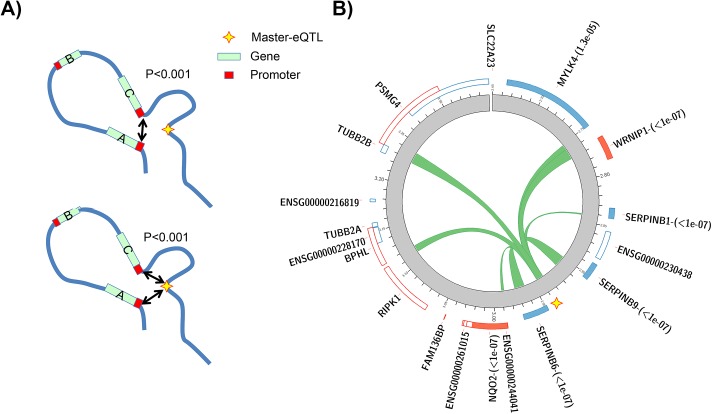
Master-eQTLs and chromatin looping (A) Circular permutations were performed to test whether genes sharing a master-eQTL are significantly more likely to loop towards one another (top) and towards the regulatory site they share (bottom—p values derived from circular permutations as described in the methods and as illustrated in S4 and S5 Figs) (B) Chromatin looping between a master-eQTL and regulated genes at the SERPINB locus on chromosome 6. Chromatin looping between the master-eQTL region and gene promoters, as defined by the Chi-C experiment, are indicated as green ribbons within the circle. Genes tested for an association on the negative (blue, orientated counter-clockwise) and positive (red, orientated clockwise) strands are shown, with those with a significant association indicated by full shading. Corresponding q values are shown after each gene’s name.

Using this approach the rate of chromatin looping between master eQTLs and their linked genes was observed to be approximately 2.6 times higher in the real data than the average of the permutations (permutation p<0.001, Fig 5A and S4 Fig), with none of the 1000 permutations showing as much looping between co-regulated genes and shared eQTLs as observed in the real data. Some genes were observed to map to the same Hi-C chromatin domain, so that the eQTL is brought into both their proximity via one higher order chromatin loop. However, distinct chromatin looping between regions harbouring an eQTL and each of the regulated genes was also observed significantly more often than expected (S4 Fig). This suggests that co-regulated genes often appear to be brought into the vicinity of a shared eQTL via multiple distinct chromatin looping events.

The promoters of genes sharing an eQTL are also found closer together in three-dimensional chromatin space than expected by chance given their genomic distance apart, (permutation p<0.001, Fig 5A and S5 Fig). Consequently master-eQTLs and their associated genes often appear to be forming higher order multi-way chromatin interactions in the cell.

An example of a co-regulated gene cluster is shown in Fig 5B. The master-eQTL at this locus on chromosome 6 is significantly associated with the expression of 6 genes. Five of these genes show evidence of chromatin looping to the region harbouring the putative regulatory variant, with the final gene residing directly upstream of the eQTL itself. The promoters of these genes were also found to loop towards one-another in three dimensional chromatin space (S3 Table), supporting a multi-way interaction at this locus. Although, as far as we are aware, no master regulatory site has previously been described in this region containing three SERPINB genes, a locus control region has been characterised at the SERPINA cluster on chromosome 14 [19], suggesting that both SERPIN loci are potentially under the influence of master regulators. Consequently there is evidence that master-eQTLs and associated regulated genes are often forming higher order multi-way chromatin interaction modules in the cell.

### Defining the potential role of master regulators in shaping genome evolution

An active role of chromatin looping in gene co-regulation suggests that such clusters may be expected to be under evolutionary constraint to be conserved together across time; co-regulated gene pairs being disproportionately found within unusually close proximity, and over 21% being within 10kb (S6 Fig). Previous studies of co-expression in mammals have argued against co-expressed gene clusters being maintained together and that rather they have generally moved apart over evolutionary time [6]. A potential limitation of these studies may be the focus on co-expression. Using our set of gene pairs showing evidence for genetic co-regulation, we tested to see if the sharing of an eQTL and chromatin interactions are linked to the conservation of gene order across species.

**Fig 6:**
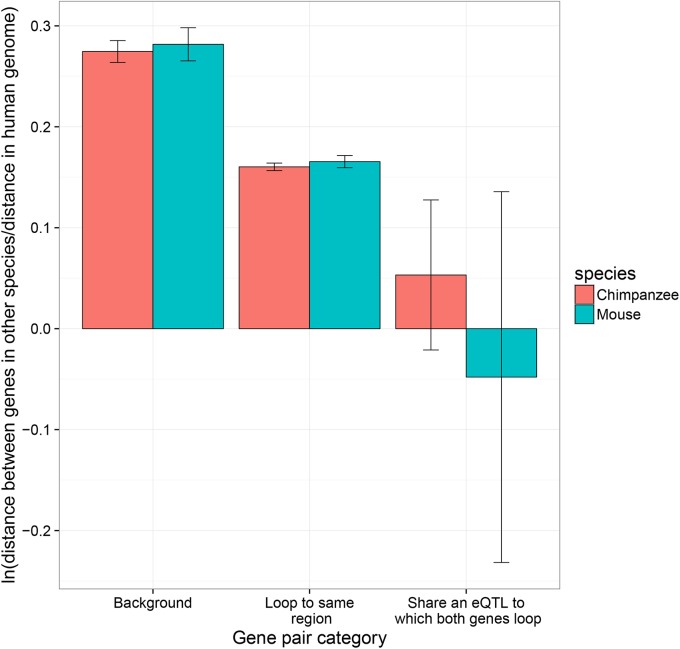
Co-regulated gene modules are linked to the conservation of inter-gene distance across evolution. For gene pairs of different categories their relative distance in the human, chimpanzee and mouse genomes were calculated. The y axis corresponding to the average (natural) log ratio of the inter-gene distances for each gene pair in each category. A higher log ratio indicates the gene pairs are, on average, generally further apart in the non-human species than in the human genome. A log ratio of 0 indicating inter-gene distance is unchanged between species. Standard errors of the mean are shown. A version of this plot including the small number of gene pairs which share an eQTL but are not linked by chromatin looping to a common region is shown in S7 Fig.

Controlling for their distance apart in the human genome, genes that loop together towards a shared genomic target were found to have been maintained at a more similar distance apart in both the chimpanzee and mouse genomes than those gene pairs with no evidence of being linked by chromatin looping (Fig 6). Where genes loop towards the same region that also harbours a shared eQTL, there appears to be an unusually strong constraint on their inter-gene distance. The average change in inter-gene distance of such gene pairs is not significantly different from 0 in both the human and chimpanzee analyses. Despite the general increase in inter-gene distance of other gene pairs, these genes linked by chromatin looping to a shared regulatory site have been maintained at largely the same genomic distance apart across these comparatively long evolutionary timescales (Fig 6). Comparing these gene pair groups to each other while controlling for their distance apart in the human genome via multiple linear regression, reaffirms gene pairs linked by chromatin looping to a shared eQTL display the smallest increases in inter-gene distance (Table 1). Chromatin looping, that has been shown to be relatively well conserved across mammals [20], appears to be associated with constraints on genome evolution. However, the combination of chromatin interactions and shared eQTLs is associated with the strongest conservation of inter-gene distances across species. Co-regulated gene modules linked by chromatin looping and a master-eQTL appear to be under unusually strong constraint to maintain them together over evolutionary time.

**Table 1:** Conservation of inter-gene distance between gene pairs sharing master-eQTLs.

	Dependent variable—ln(distance apart in other species + 1)	
	Chimpanzee	Mouse
**ln(Human distance apart + 1)**	0.92 (0.003)***	0.97 (0.005)***
**Genes loop to same region (chimpanzee n = 60478, mouse = 55218)**	-0.08 (0.010)***	-0.10 (0.015)***
**Genes share eQTL but not linked by looping (chimpanzee n = 71, mouse = 63)**	-0.13 (0.12)	-0.18 (0.19)
**Genes loop to same region harbouring shared eQTL (chimpanzee n = 214, mouse = 176)**	-0.26 (0.068)**	-0.34 (0.11)**
**Other gene pairs (background pairs) (chimpanzee n = 13039, mouse = 11961)**	NA (Reference)	NA (Reference)
**Constant**	1.18 (0.036)***	0.68 (0.056)***
**Observations**	73,802	67,418

*p<0.05

**p<0.005

***p<5x10^-4^

Multiple linear regression coefficients, and standard errors in brackets, when testing whether genes linked by chromatin looping and/or a shared eQTL are found close together in other mammals having controlled for their corresponding distance apart in the human genome. Each gene pair type was tested relative to the background set of gene pairs that neither show evidence of looping towards a common site or of sharing an eQTL. A significant negative coefficient indicates the corresponding group of gene pairs are generally found closer together in the non-human species than gene pairs in this background set, when controlling for their observed distance apart in the human genome. Results where each group are compared to the set of gene pairs looping to the same region but that do not share an eQTL are shown in S4 Table.

### Potential role of shared regulatory sites in pleiotropy

Shared regulatory sites may be expected to be particularly important in shaping downstream phenotypes due to their potential link to multiple biological pathways. Previous studies have shown that eVariants are also often a GWAS variant [12], but whether master-eQTLs are more likely to be linked to multiple phenotypes has not previously been examined. To investigate this we first identified the total set of variants in LD with each eQTL at different thresholds (R^2^ > = 0.6, > = 0.8, = 1), and using information from the GWAS catalogue [21] determined the total number of phenotypes linked to each set. Where an eQTL was in LD with multiple GWAS variants associated with the same or similar phenotype they were collapsed into one, providing a non-redundant count of phenotypes linked to the variants in LD with each eQTL. Depending on the LD threshold used, master-eQTLs were observed to be between 2.5 and 2.6 times more likely to be linked to one or more GWAS phenotype than eQTLs linked to only one gene (chi-squared p < 5x10^-5^ at all three LD thresholds). Notably, this broadly tallies with master-eQTLs being associated with 2.34 genes on average.

**Fig 7:**
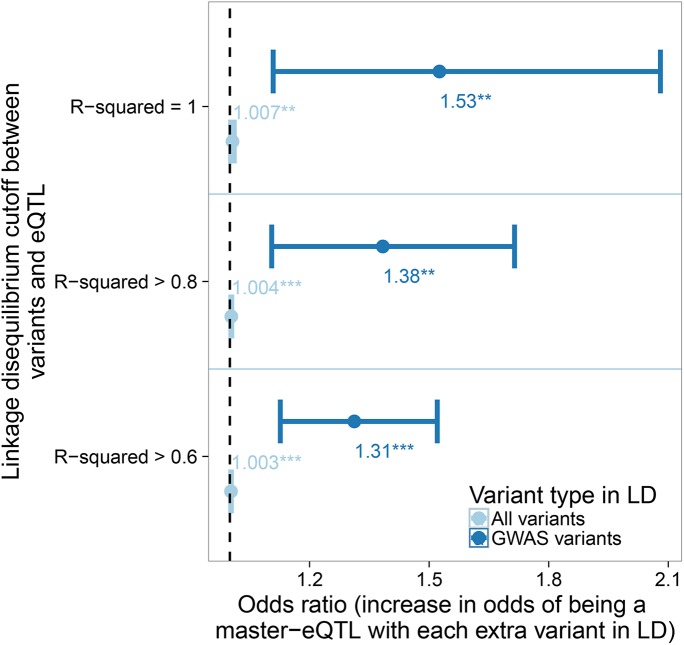
The increase in odds of an eQTL being linked to multiple genes given each extra non-redundant GWAS variant found in LD. The more distinct GWAS phenotypes associated with variants in LD, the greater the odds an eQTL is a master-eQTL (dark blue). Odds ratio and 95% confidence intervals were calculated using logistic regression and the number of all variants within the same LD threshold was fitted as a covariate (light blue) to control for any potential confounding due to master-eQTLs being in regions of high LD (** p < 0.01, *** p < 0.001).

Multi-eQTLs were observed to be associated with a modest, albeit significant, increase in the number of variants in LD (Fig 7). However, accounting for this general enrichment of linked variants using logistic regression, the sets of variants in LD with master-eQTLs were found to be generally associated with more distinct phenotypes than variants in LD with single-eQTLs. Each extra phenotype with which an eQTL is associated, increases the odds of it being a multi-eQTL by a factor of between 1.31 to 1.51 depending on the LD threshold used (Fig 7). Amongst all eQTLs there is a significant correlation between the number of genes whose expression it is linked to and the number of non-redundant GWAS variants in LD (R^2^ threshold of > = 0.6. Partial correlation Kendall’s tau: 0.094; p = 4.34x10^-21^ when accounting for the total number of variants in LD with each eQTL). Consequently eQTLs linked to multiple genes are more likely to be in LD with multiple, non-redundant GWAS variants suggesting the regions of shared regulatory sites are key hubs of disease risk and human phenotypes.

## Discussion

There is an increasing focus on understanding the genetics of gene regulation, with studies such as GTEx [13] assaying the links between genetic variants and gene expression in hundreds of individuals and across multiple tissues. Despite the inarguable utility of these analyses, their focus on producing lists of individual gene to individual variant associations perhaps oversimplifies genetic regulation.

In this study we identify independent cis-eQTLs and show that the sharing of non-redundant regulatory variants is a relatively common phenomenon. Shared regulatory variants are not restricted to a small handful of sites, but found across over 10% of all pruned regulatory sites. Although distinguishing between a single master regulatory site, and multiple regulatory sites in perfect, or near perfect, LD is difficult [22], suggesting this estimate is likely an upper bound, we have shown that genes sharing a master-eQTL show correlated changes in expression linked to the variant’s genotype across tissues. This therefore supports the idea of substantial levels of co-regulation in the human genome. Likewise the patterns of chromatin looping linked to master-eQTLs adds further support to the function of these sites.

The observation that genes sharing a regulatory site are less likely to move apart over evolutionary time also suggests that regulatory site sharing is not simply a result of regulation “leaking” from one gene to its neighbours as has previously been suggested [6]. Selection is expected to break up gene pairs if regulation of one is adversely affecting its neighbours [6]. In contrast, such gene groups are unusually well conserved, suggesting the opposite is likely to be the case, and that their proximity is being maintained over relatively long evolutionary timescales.

A limitation of eQTL studies is that they are dependent on a polymorphism falling within the regulatory site. Many, if not the majority, of regulatory sites are therefore likely to be missed in any study based on genetic regulatory variants. However, we can extrapolate from the numbers observed in this study. Although we identified just 534 shared eQTLs, only 3422 genes were linked to an eQTL in this analysis, with the majority of regulatory sites unlikely to be tagged by an eQTL. With as many as 50,000 (coding and non-coding) genes in the genome [23], this suggests there may be several thousand shared regulatory sites in total.

As defining the locations of eQTLs is a common post-GWAS prioritisation approach, understanding the distribution and location of these sites has important implications for interpreting disease loci. This study has shown that master-eQTLs are more likely to be linked to several phenotypes than other eQTLs. Pleiotropy is receiving increasing attention, in part due to its potential for increasing power in association studies [22], and these results provide a potential mechanism for interpreting pleiotropy outside the most commonly studied coding regions.

## Methods

### Ethics statement

Ethical, legal and social implication (ELSI) statements for the GEUVADIS and GTEx datasets used in this study can be found at: http://www.geuvadis.org/web/geuvadis/resources/elsi and https://www.ncbi.nlm.nih.gov/pmc/articles/PMC4010069/#S11title.

### Defining independent eQTLs

Cis-eVariants were defined using the 379 European Individuals in the 1000 genomes project that have matching GEUVADIS gene expression data available [12]. All 1000 genomes phase 3 variants [24] within 500kb of the transcription start site of each gene were first tested for an association with the gene’s expression levels using linear regression, fitting sex and 1000 genomes population of origin as covariates. Permutation p values were then calculated for those cis-eVariants reaching a nominal significance of p < 0.00001 using the lmPerm R package. A maximum of 1 million iterations were run for each variant, with iterations terminating if the estimated standard deviation of the p value fell below 0.1 of the estimated p value [25].

Forward stepwise regression was used to reduce the redundancy among these variants and identify conditionally independent eQTLs. The lmPerm R package again was used to determine conditioned permutation p values (with a maximum iteration number of 10 million). As these independent eVariants, each expected to correspond to a distinct eQTL, were defined for each gene independently, two different variants could be associated with different genes, despite being redundant and tagging the same functional variant. To try and remove this redundancy, a final step was undertaken where redundant eQTLs were merged across genes. For a given gene, gene x, we determined those eVariants that were dropped following pruning but that corresponded to the same eVariant as an eQTL that survived pruning for another gene, gene y. If this eVariant was equally effective at explaining variation in gene x’s gene expression as the current eQTL of gene x, the two variants were marked as potentially redundant (if an ANOVA F-test p value comparing linear models with both or just one SNP fitted against the gene’s expression was > 0.05). The variant with the larger chromatin enrichment in the immediate vicinity (see below for calculation) was subsequently chosen to be the eQTL assigned to both genes x and y if equally effective in explaining each gene’s variation in expression. In total 7.8% of non-redundant eVariants were replaced with another representative in this way. A final round of forward regression with this final set of pruned eVariants was undertaken to ensure this process did not affect the redundancy among eQTLs. Finally any eVariants not in V6 of GTEx were excluded from all analyses, leaving the final set of 5254 non-redundant eQTLs. A comparison of the numbers of eQTLs linked to multiple genes prior to and after this final merging step is shown in S8 Fig.

### Calculating the expected overlap between eQTLs

To determine the expected amount of eQTL sharing among genes purely by chance we determined the frequency at which variants of a particular minor allele frequency (MAF, grouped into 2% frequency bins) and distance from the TSS of the respective gene (10kb bins) were called an independent eVariant. These frequencies equate to the probability of a variant corresponding to an eQTL of a gene given its MAF and distance to the gene’s TSS. When calculating these frequencies we also conservatively included all eVariants in perfect LD with each independent eQTL, to account for any increased eQTL sharing resulting from the same variant being picked for each gene when multiple variants were of equal significance. These frequencies were used in 100 permutations to assess the expected amount of eQTL sharing by chance, based purely on this observed distribution of regulatory variants in the genome. In each permutation all variants in the final set of eQTLs were first randomly assigned to just one of the genes of which it was an eQTL. Using the probabilities in S2 Fig the probability of this eQTL being associated with each of the remaining genes within 500kb was then determined. If this probability was greater than a randomly drawn number from a uniform distribution between 0 and 1, this variant was deemed to be an eQTL for the corresponding gene in this permutation. In this way the frequency of observing eQTLs linked to multiple genes based on their minor allele frequency and distance to nearby genes could be determined. The average number and 95% interval ranges of eQTLs linked to different numbers of genes across permutations was finally determined to compare to the observed counts in Fig 3.

In our approach to define master-eQTLs described above we had undertaken a final step merging redundant eQTLs across genes. This had the effect of increasing eQTL sharing. However, as shown in S8 Fig, even without this final merging step eQTL sharing was substantially higher than expected based purely on the distribution of eQTLs in the genome represented in S2 Fig. Consequently, even without collapsing eVariants in high LD into one eQTL, there is an enrichment of eQTL sharing in the genome.

### Comparison to GTEx derived eQTLs

To test the cross-dataset and cross-tissue reproducibility of eVariants (i.e. whether the eVariants are significant in other tissues), GTEx P values for the eVariants defined in this study were obtained from http://www.gtexportal.org/home/. As discussed any variant not tested by GTEx was excluded from all analyses.

To test whether the defined master-eQTLs demonstrate unusually high correlations between their size and direction of effects on genes across tissues, we also obtained GTEx eQTL coefficients from the GTEx website. These coefficients represent the degree to which the gene’s expression changes upon changes in the eVariant’s genotype. Consequently a high correlation (r_tiss_) between two sets of coefficients suggests the expression of the two corresponding genes changes in a similar way across tissues upon changes in the genotype of the eVariant(s). In this analysis we calculated the Pearson’s correlation (r_tiss_) between all sets of coefficients associated with our pruned eQTLs having excluded eQTLs for which a coefficient was not available in at least three quarters of the 44 tissues studied. These correlations therefore represent the degree to which two different eQTL-gene pairs show similar sizes and direction of effects across tissues (see examples in Fig 4A and 4B). From the total set of correlations a subset corresponding to the correlations between the coefficients associated with a master-eQTL and two associated genes were first extracted (“Share a master eQTL” group). The remaining group (“others”) was composed of those correlations where the eQTL was not tagged by the same variant i.e. the two sets of coefficients corresponded to two different eQTL-gene pairs. We then sampled 100 times from this group subsets of correlations corresponding to pairs of genes whose inter-gene distances approximately matched (same 50kb bin) the distribution of inter-gene distances of genes sharing a master-eQTL (“close proximity” group). To investigate the link between LD and the correlations between these tissue-level coefficients we calculated LD between all pairs of independent eQTL variants within 500kb of each other using PLINK [26].

### Chromatin looping

Interacting regions of the human genome were obtained from Jin et al. [16] and Mifsud et al. [17]. The former is a genome-wide study of looping in the human genome and the latter a higher resolution study of loops specifically associated with gene promoters.

To assess the significance of the numbers of eQTLs and gene pairs found within genomic regions interacting in the genome-wide study of Jin et al. [16] we adopted a circular permutation approach. All autosomes being concatenated and in each permutation all genomic regions defined in the Hi-C dataset being moved the same random distance along the genome (shifts being between 20kb and the total length of all the autosomes). Any regions falling off the end of the concatenated genome were added back on to the beginning. In this way we maintained the relationship between genomic regions, as well as their relative distances and sizes, but broke their relationship with eQTLs and gene locations. If the relationship between eQTLs and the TSS they are linked to is unrelated to chromatin interactions, we would have frequently expected to see as many eQTLs and TSSs linked by chromatin looping in these permuted Hi-C datasets. Our (one tailed) p value was calculated as the proportion of permutations where the number of eQTL-gene pair associations linked by Hi-C loops was greater than or equal to the number in the unpermuted data. Links between TSS pairs were assessed in the same way i.e. the Hi-C dataset was shifted a random distance along the circularised genome and the proportion of permutations determined where the number of gene pairs linked by chromatin looping was greater than or equal to the number in the real data.

### Genome evolution

The orthologues of human genes in the mouse and chimpanzee genomes were obtained from Ensembl [23]. Only orthologues with a one to one relationship between species were kept. All inter-gene distances were calculated between the TSS of genes found on the same chromosome in both species i.e. the measure is the change in distance between genes due to insertions, deletions, inversions etc. but not translocations.

### Master eQTLs and disease

LD levels between independent eQTL variants defined in this study and all variants within 500kb were calculated using the 1000 genomes phase 3 European genotype dataset [24] and VCFtools [27]. The GWAS catalogue [21] was used to determine the number of phenotypes that have been associated with the set of variants in LD with each eQTL, and redundancy among phenotypes was removed by manually assessing the phenotypes linked to each eQTL and collapsing duplicates or analogous phenotypes (e.g. weight and BMI). Logistic regression was used to test for an association between master-eQTLs and their associated number of phenotypes. The response variable being 0 for eQTLs linked to only one gene and 1 for master-eQTLs. The total number of variants in LD with the corresponding eQTL was fitted as a covariate alongside the total number of associated phenotypes to account for any differences in the extent of LD around master-eQTLs and single gene eQTLs.

## Supporting information

S1 Fig Schematic of confounding of eQTLs by linkage disequilibrium. Each point represents a single genetic variant along a genomic region and their log-transformed P value in two different tissues. At a region containing two causative regulatory variants that act in a tissue-specific manner (red and blue dots), LD can increase the apparent replication of eQTLs. Polymorphisms between the causative variants in this example appearing to replicate in both tissues due to being in LD with both causative variants. Ideally, following pruning, just the causative regulatory variant in the respective tissue will be selected. This should have little impact on replication within different datasets derived from the same tissue type, but will lead to a decrease in the replication of eQTLs across tissues. Variants showing spurious cross-tissue replication removed during pruning. Randomly selected eVariants on the other hand will often appear to replicate in this example, and their p value is expected to be close to the median of all p values in the region, meaning subsampling random sets of eVariants will have little impact on the genome-wide median log transformed p value compared to the total set. Consequently the limited impact of pruning on reproducibility in the same tissue type but decreased reproducibility in different tissues that is observed in Fig 1 is consistent with pruning reducing such spurious cross-tissue reproducibility. Randomly selected variants expecting to show little change in reproducibility as illustrated by the grey area in the lower panel of Fig 1.
(PDF)

S2 Fig Frequency of variants being independent eVariants, or in perfect LD with an independent eVariant, as a function of their allele frequency and distance to gene. The legend indicates the midpoint of the corresponding 2% minor allele frequency bin.
(PDF)

S3 Fig Number of gene pairs found to both loop towards a shared eQTL. Total numbers of pairs identified using each dataset are shown in brackets, with 33% of the Hi-C pairs common to the CHi-C dataset.
(TIF)

S4 Fig Enrichment of chromatin interactions between master-eQTLs and linked gene pairs. The distributions of the number of gene pairs linked to their shared eQTL via chromatin looping in 1000 circular permutations of the Hi-C data. Results where two different chromatin interactions link the genes to the eQTL (different HiC targets) and where the two genes are linked to the eQTL via one chromatin loop interaction (same HiC target) are shown. The corresponding observed numbers in the real, unpermuted data are indicated by vertical lines (same HiC target permutation p = 0.003, different HiC target p = 0.012, combined p < 0.001).
(PNG)

S5 Fig Looping between promoters of genes sharing an eQTL. The number of gene pairs sharing an eQTL observed to loop together in the real unpermuted data (vertical line) was higher than observed following all of the HiC data permutations.
(PNG)

S6 Fig The inter-gene distance of gene pairs linked by a shared eQTL and the theoretical background distribution of inter-gene distances. The background distribution is the intergene distance of all gene pairs tested for an association with one of the non-redundant eQTLs.
(PDF)

S7 Fig Version of Fig 6 with the small group of genes that share an eQTL but do not show evidence of looping to the same region included. (PDF)

S8 Fig Observed and expected number of eQTLs linked to different numbers of genes. The observed number without the subsequent grouping step is shown i.e. without grouping eQTLs across genes that were redundant.
(PDF)

S1 Table The final non-redundant set of eVariants used in this study. (XLSX)

S2 Table Gene pairs that share a master-eQTL to which both genes loop towards. (XLSX)

S3 Table Gene pairs that share a master-eQTL and whose TSSs are linked by chromatin looping. (XLSX)

S4 Table Reanalysis of Table 1 with the reference gene pair group changed to those gene pairs looping towards a shared genomic region but that do not share a known eQTL. (XLSX)

## References

[1] Grosveld F, van Assendelft GB, Greaves DR, Kollias G. Position-independent, high-level expression of the human beta-globin gene in transgenic mice. Cell. 1987;51: 975–985. 3690667 10.1016/0092-8674(87)90584-8

[2] Li Q, Peterson KR, Fang X, Stamatoyannopoulos G. Locus control regions. Blood. 2002;100: 3077–3086. 10.1182/blood-2002-04-1104 12384402 PMC2811695

[3] Albert FW, Kruglyak L. The role of regulatory variation in complex traits and disease. Nat Rev Genet. 2015;16: 197–212. 10.1038/nrg3891 25707927

[4] Mohammadi P, Castel SE, Brown AA, Lappalainen T. Quantifying the regulatory effect size of cis-acting genetic variation using allelic fold change. bioRxiv. 2016; 078717. 10.1101/gr.216747.116 PMC5668944 29021289

[5] Hurst LD, Pál C, Lercher MJ. The evolutionary dynamics of eukaryotic gene order. Nat Rev Genet. 2004;5: 299–310. 10.1038/nrg1319 15131653

[6] Liao B-Y, Zhang J. Coexpression of Linked Genes in Mammalian Genomes Is Generally Disadvantageous. Mol Biol Evol. 2008;25: 1555–1565. 10.1093/molbev/msn101 18440951 PMC2734128

[7] Singer GAC, Lloyd AT, Huminiecki LB, Wolfe KH. Clusters of Co-expressed Genes in Mammalian Genomes Are Conserved by Natural Selection. Mol Biol Evol. 2005;22: 767–775. 10.1093/molbev/msi062 15574806

[8] Weber CC, Hurst LD. Support for multiple classes of local expression clusters in Drosophila melanogaster, but no evidence for gene order conservation. Genome Biol. 2011;12: R23 10.1186/gb-2011-12-3-r23 21414197 PMC3129673

[9] Li W, Notani D, Rosenfeld MG. Enhancers as non-coding RNA transcription units: recent insights and future perspectives. Nat Rev Genet. 2016;17: 207–223. 10.1038/nrg.2016.4 26948815

[10] Sivakumaran S, Agakov F, Theodoratou E, Prendergast JG, Zgaga L, Manolio T, et al Abundant Pleiotropy in Human Complex Diseases and Traits. Am J Hum Genet. 2011;89: 607–618. 10.1016/j.ajhg.2011.10.004 22077970 PMC3213397

[11] Hindorff LA, Sethupathy P, Junkins HA, Ramos EM, Mehta JP, Collins FS, et al Potential etiologic and functional implications of genome-wide association loci for human diseases and traits. Proc Natl Acad Sci U S A. 2009;106: 9362–9367. 10.1073/pnas.0903103106 19474294 PMC2687147

[12] Lappalainen T, Sammeth M, Friedländer MR, Hoen PAC ‘t, Monlong J, Rivas MA, et al Transcriptome and genome sequencing uncovers functional variation in humans. Nature. 2013;501: 506–511. 10.1038/nature12531 24037378 PMC3918453

[13] Consortium TGte, Ardlie KG, Deluca DS, Segrè AV, Sullivan TJ, Young TR, et al The Genotype-Tissue Expression (GTEx) pilot analysis: Multitissue gene regulation in humans. Science. 2015;348: 648–660. 10.1126/science.1262110 25954001 PMC4547484

[14] Peltekova VD, Lemire M, Qazi AM, Zaidi SHE, Trinh QM, Bielecki R, et al Identification of genes expressed by immune cells of the colon that are regulated by colorectal cancer-associated variants. Int J Cancer. 2014;134: 2330–2341. 10.1002/ijc.28557 24154973 PMC3949167

[15] Jeziorska DM, Jordan KW, Vance KW. A systems biology approach to understanding cis-regulatory module function. Semin Cell Dev Biol. 2009;20: 856–862. 10.1016/j.semcdb.2009.07.007 19660565

[16] Jin F, Li Y, Dixon JR, Selvaraj S, Ye Z, Lee AY, et al A high-resolution map of the three-dimensional chromatin interactome in human cells. Nature. 2013;503: 290–294. 10.1038/nature12644 24141950 PMC3838900

[17] Mifsud B, Tavares-Cadete F, Young AN, Sugar R, Schoenfelder S, Ferreira L, et al Mapping long-range promoter contacts in human cells with high-resolution capture Hi-C. Nat Genet. 2015;47: 598–606. 10.1038/ng.3286 25938943

[18] Cabrera CP, Navarro P, Huffman JE, Wright AF, Hayward C, Campbell H, et al Uncovering networks from genome-wide association studies via circular genomic permutation. G3 Bethesda Md. 2012;2: 1067–1075. 10.1534/g3.112.002618 PMC3429921 22973544

[19] Namciu SJ, Friedman RD, Marsden MD, Sarausad LM, Jasoni CL, Fournier REK. Sequence organization and matrix attachment regions of the human serine protease inhibitor gene cluster at 14q32.1. Mamm Genome Off J Int Mamm Genome Soc. 2004;15: 162–178. 10.1007/s00335-003-2311-y 15014966

[20] Vietri Rudan M, Barrington C, Henderson S, Ernst C, Odom DT, Tanay A, et al Comparative Hi-C reveals that CTCF underlies evolution of chromosomal domain architecture. Cell Rep. 2015;10: 1297–1309. 10.1016/j.celrep.2015.02.004 25732821 PMC4542312

[21] Welter D, MacArthur J, Morales J, Burdett T, Hall P, Junkins H, et al The NHGRI GWAS Catalog, a curated resource of SNP-trait associations. Nucleic Acids Res. 2014;42: D1001–1006. 10.1093/nar/gkt1229 24316577 PMC3965119

[22] Solovieff N, Cotsapas C, Lee PH, Purcell SM, Smoller JW. Pleiotropy in complex traits: challenges and strategies. Nat Rev Genet. 2013;14: 483–495. 10.1038/nrg3461 23752797 PMC4104202

[23] Flicek P, Amode MR, Barrell D, Beal K, Billis K, Brent S, et al Ensembl 2014. Nucleic Acids Res. 2014;42: D749–D755. 10.1093/nar/gkt1196 24316576 PMC3964975

[24] 1000 Genomes Project Consortium, Abecasis GR, Altshuler D, Auton A, Brooks LD, Durbin RM, et al A map of human genome variation from population-scale sequencing. Nature. 2010;467: 1061–1073. 10.1038/nature09534 20981092 PMC3042601

[25] Wheeler B, Torchiano M. lmPerm: Permutation Tests for Linear Models [Internet]. 2016. Available: https://cran.r-project.org/web/packages/lmPerm/index.html

[26] Purcell S, Neale B, Todd-Brown K, Thomas L, Ferreira MAR, Bender D, et al PLINK: a tool set for whole-genome association and population-based linkage analyses. Am J Hum Genet. 2007;81: 559–575. 10.1086/519795 17701901 PMC1950838

[27] Danecek P, Auton A, Abecasis G, Albers CA, Banks E, DePristo MA, et al The variant call format and VCFtools. Bioinforma Oxf Engl. 2011;27: 2156–2158. 10.1093/bioinformatics/btr330 PMC3137218 21653522

[28] Tenesa A, Farrington SM, Prendergast JGD, Porteous ME, Walker M, Haq N, et al Genome-wide association scan identifies a colorectal cancer susceptibility locus on 11q23 and replicates risk loci at 8q24 and 18q21. Nat Genet. 2008;40: 631–637. 10.1038/ng.133 18372901 PMC2778004

