# Supplementary material for: Shared regulatory sites are abundant in the human genome and shed light on genome evolution and disease pleiotropy
Source: PLoS Genet. 2017 Mar 10;13(3):e1006673. doi: 10.1371/journal.pgen.1006673 (PMC5365138; doi:10.1371/journal.pgen.1006673)
Supplement: S1 Fig — Each point represents a single genetic variant along a genomic region and their log-transformed P value in two different tissues. At a region containing two causative regulatory variants that act in a tissue-specific manner (red and blue dots), LD can increase the apparent replication of eQTLs. Polymorphisms between the causative variants in this example appearing to replicate in both tissues due to being in LD with both causative variants. Ideally, following pruning, just the causative regulatory variant in the respective tissue will be selected. This should have little impact on replication within different datasets derived from the same tissue type, but will lead to a decrease in the replication of eQTLs across tissues. Variants showing spurious cross-tissue replication removed during pruning. Randomly selected eVariants on the other hand will often appear to replicate in this example, and their p value is expected to be close to the median of all p values in the region, meaning subsampling random sets of eVariants will have little impact on the genome-wide median log transformed p value compared to the total set. Consequently the limited impact of pruning on reproducibility in the same tissue type but decreased reproducibility in different tissues that is observed in Fig 1 is consistent with pruning reducing such spurious cross-tissue reproducibility. Randomly selected variants expecting to show little change in reproducibility as illustrated by the grey area in the lower panel of Fig 1. (PDF) [file pgen.1006673.s001.pdf]

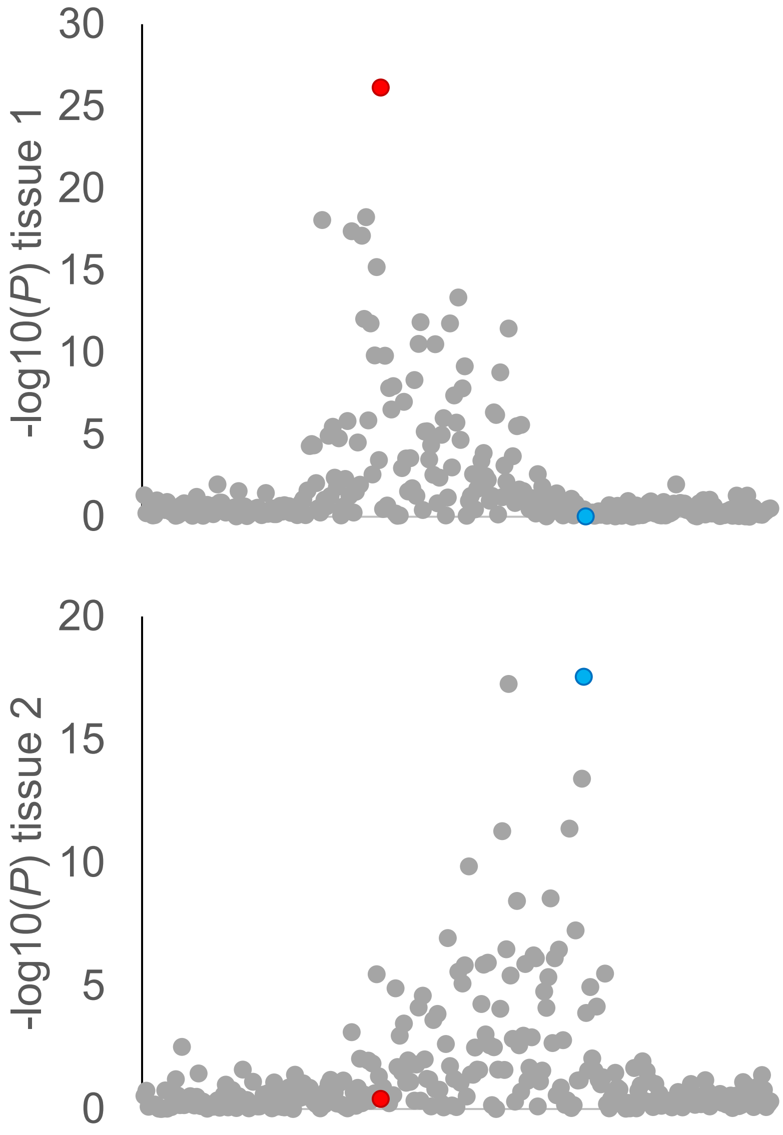

Supplementary Figure 1. Schematic of confounding of eQTLs by linkage disequilibrium. Each point represents a single genetic variant along a genomic region and their log-transformed P value in two different tissues. At a region containing two causative regulatory variants that act in a tissue-specific manner (red and blue dots), LD can increase the apparent replication of eQTLs. Polymorphisms between the causative variants in this example appearing to replicate in both tissues due to being in LD with both causative variants. Ideally, following pruning, just the causative regulatory variant in the respective tissue will be selected. This should have little impact on replication within different datasets derived from the same tissue type, but will lead to a decrease in the replication of eQTLs across tissues. Variants showing spurious cross-tissue replication being removed. Randomly selected eVariants on the other hand will often appear to replicate in this example, and their p value is expected to be close to the median of all p values in the region, meaning subsampling random sets of eVariants will have little impact on the genome-wide median log transformed p value compared to the total set. Consequently the limited impact of pruning on reproducibility in the same tissue type but decreased reproducibility in different tissues that is observed in Figure 1 is consistent with pruning reducing such spurious cross-tissue reproducibility. Randomly selected variants expecting to show little change in reproducibility as illustrated by the grey area in the lower panel of Figure 1.
