# Supplementary material for: Shared regulatory sites are abundant in the human genome and shed light on genome evolution and disease pleiotropy
Source: PLoS Genet. 2017 Mar 10;13(3):e1006673. doi: 10.1371/journal.pgen.1006673 (PMC5365138; doi:10.1371/journal.pgen.1006673)
Supplement: S2 Fig — The legend indicates the midpoint of the corresponding 2% minor allele frequency bin. (PDF) [file pgen.1006673.s002.pdf]

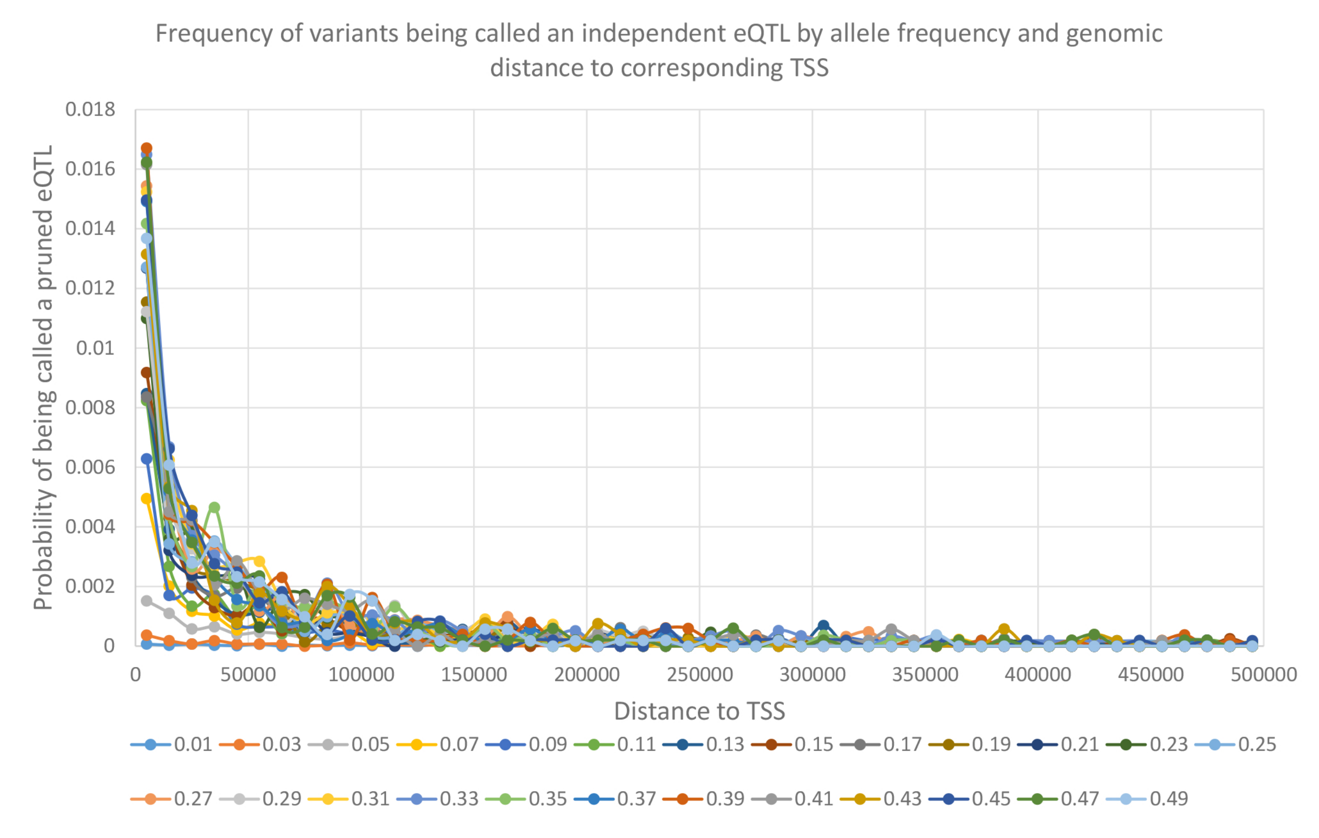
Frequency of variants being called an independent eQTL by allele frequency and genomic distance to corresponding TSS
Probability of being called a pruned eQTL
0.018
0.016
0.014
0.012
0.01
0.008
0.006
0.004
0.002
0
0
50000
100000
150000
200000
250000
300000
350000
400000
450000
500000
Distance to TSS
0.01
0.03
0.05
0.07
0.09
0.11
0.13
0.15
0.17
0.19
0.21
0.23
0.25
0.27
0.29
0.31
0.33
0.35
0.37
0.39
0.41
0.43
0.45
0.47
0.49
