# Supplementary figures and images for: Shared regulatory sites are abundant in the human genome and shed light on genome evolution and disease pleiotropy
Source: PLoS Genet. 2017 Mar 10;13(3):e1006673. doi: 10.1371/journal.pgen.1006673 (PMC5365138; doi:10.1371/journal.pgen.1006673)

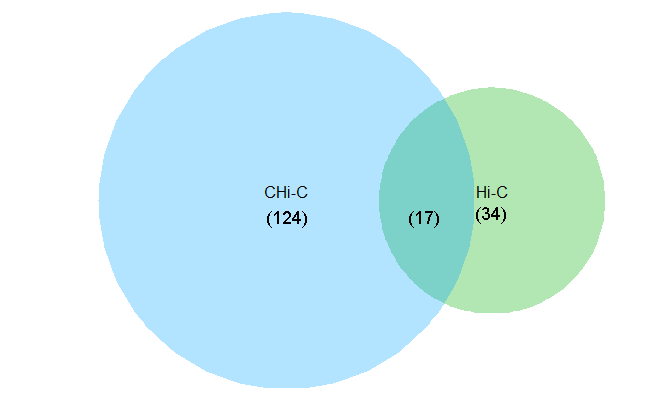

Supplement: S3 Fig — Total numbers of pairs identified using each dataset are shown in brackets, with 33% of the Hi-C pairs common to the CHi-C dataset. (TIF) [file pgen.1006673.s003.tif]

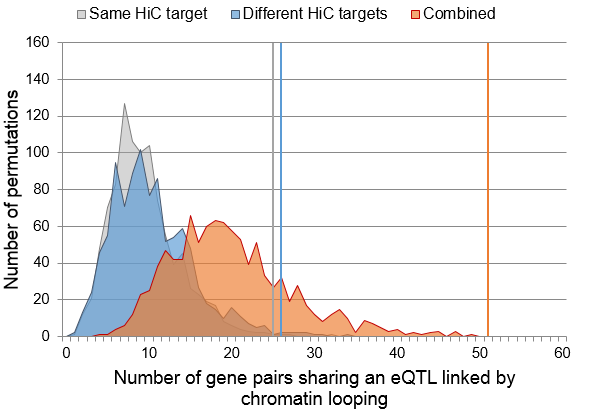

Supplement: S4 Fig — The distributions of the number of gene pairs linked to their shared eQTL via chromatin looping in 1000 circular permutations of the Hi-C data. Results where two different chromatin interactions link the genes to the eQTL (different HiC targets) and where the two genes are linked to the eQTL via one chromatin loop interaction (same HiC target) are shown. The corresponding observed numbers in the real, unpermuted data are indicated by vertical lines (same HiC target permutation p = 0.003, different HiC target p = 0.012, combined p < 0.001). (PNG) [file pgen.1006673.s004.PNG]

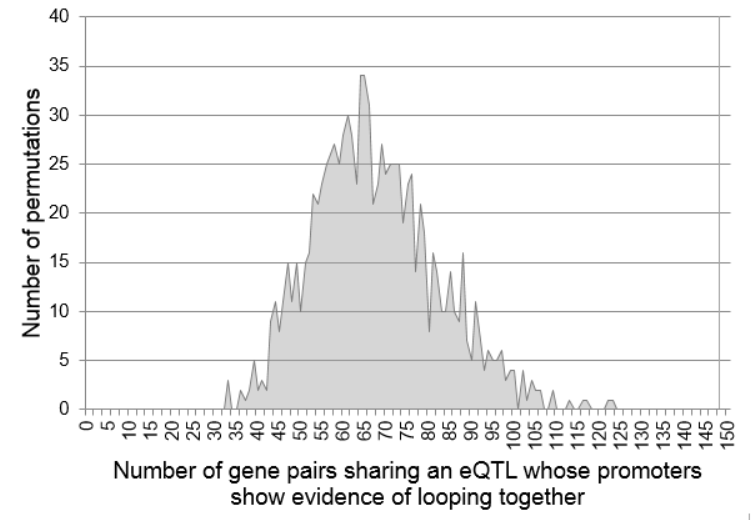

Supplement: S5 Fig — The number of gene pairs sharing an eQTL observed to loop together in the real unpermuted data (vertical line) was higher than observed following all of the HiC data permutations. (PNG) [file pgen.1006673.s005.PNG]

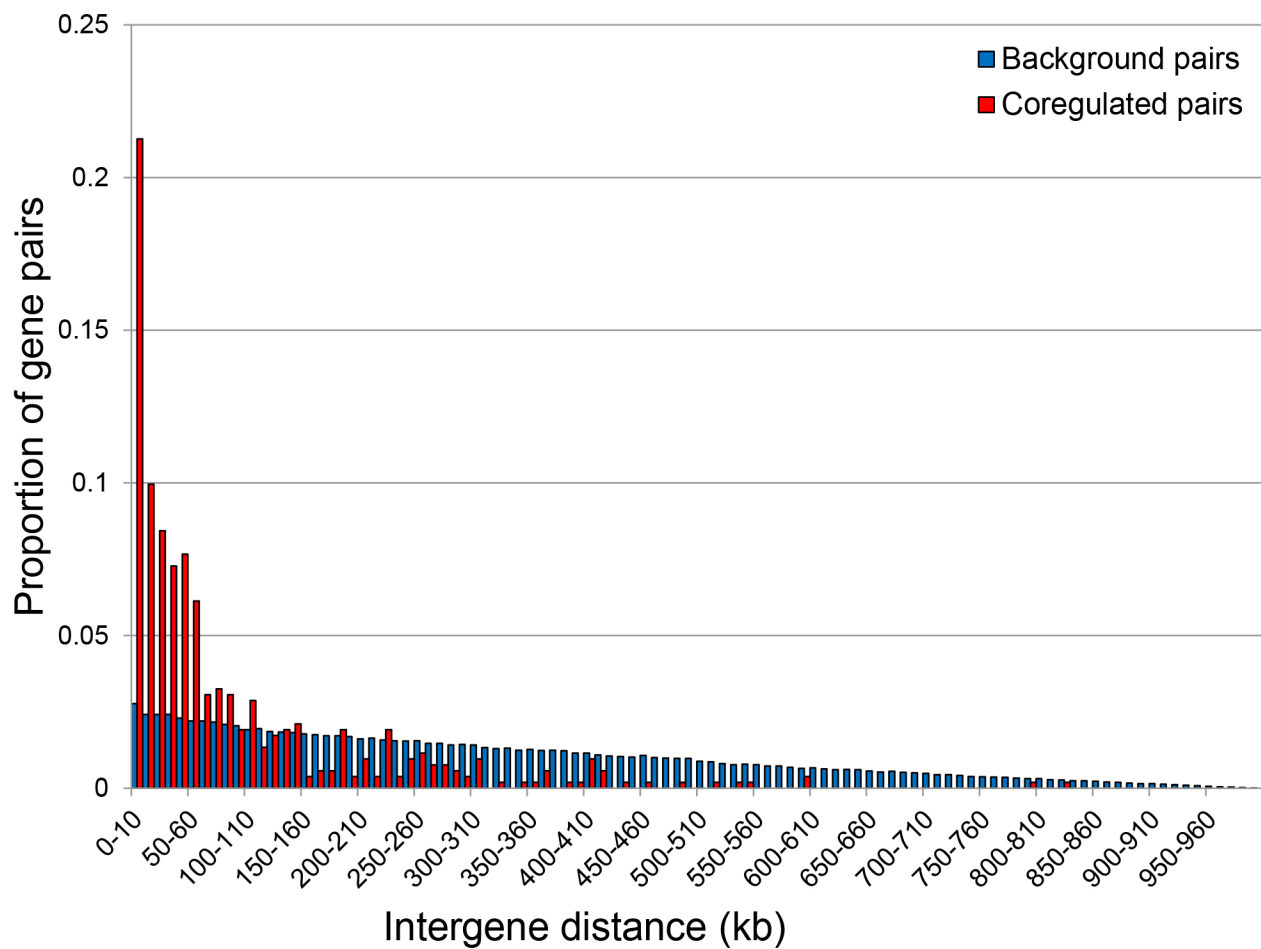

Supplement: S6 Fig — The background distribution is the intergene distance of all gene pairs tested for an association with one of the non-redundant eQTLs. (PDF) [file pgen.1006673.s006.pdf]

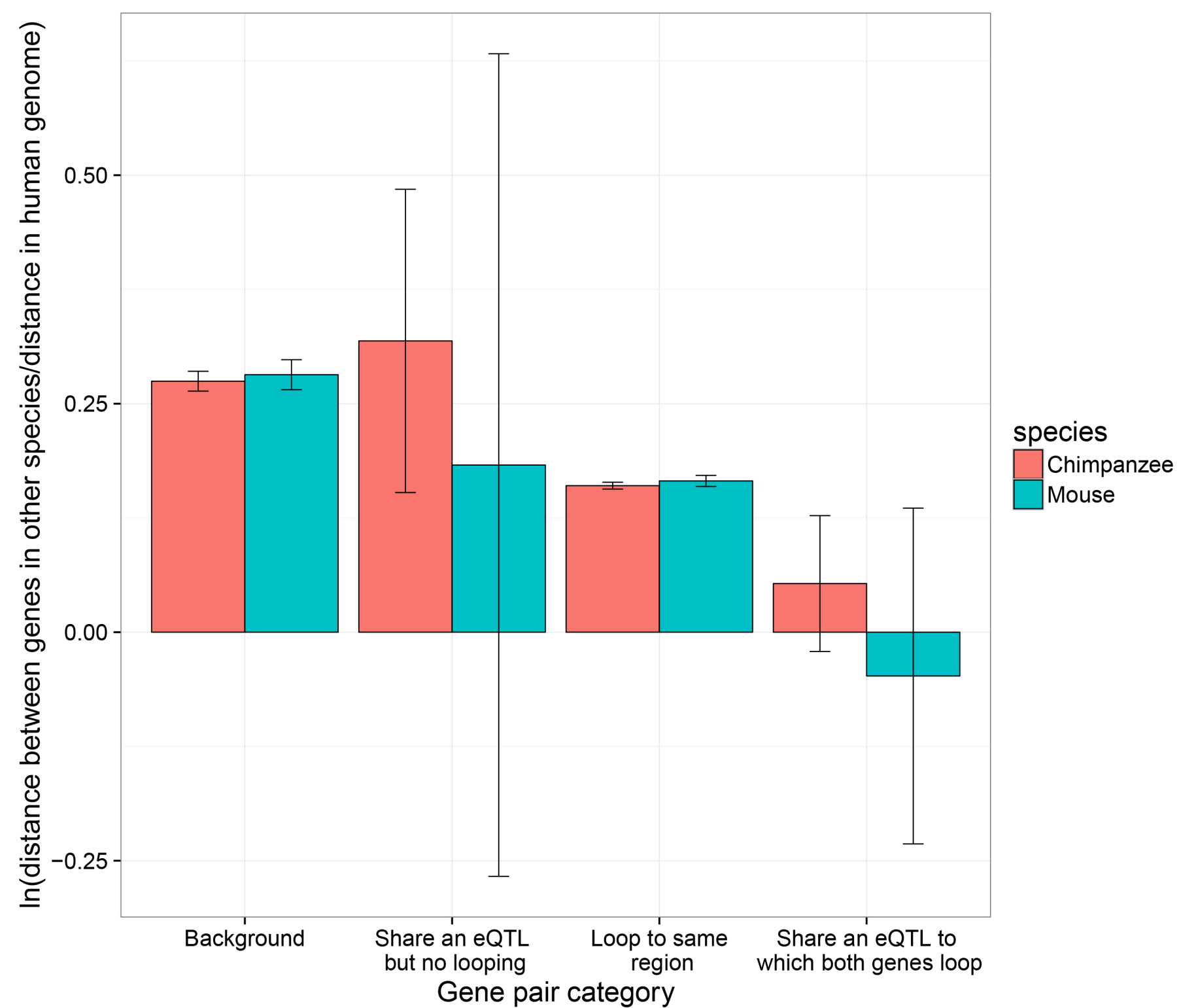

Supplement: S7 Fig — (PDF) [file pgen.1006673.s007.pdf]

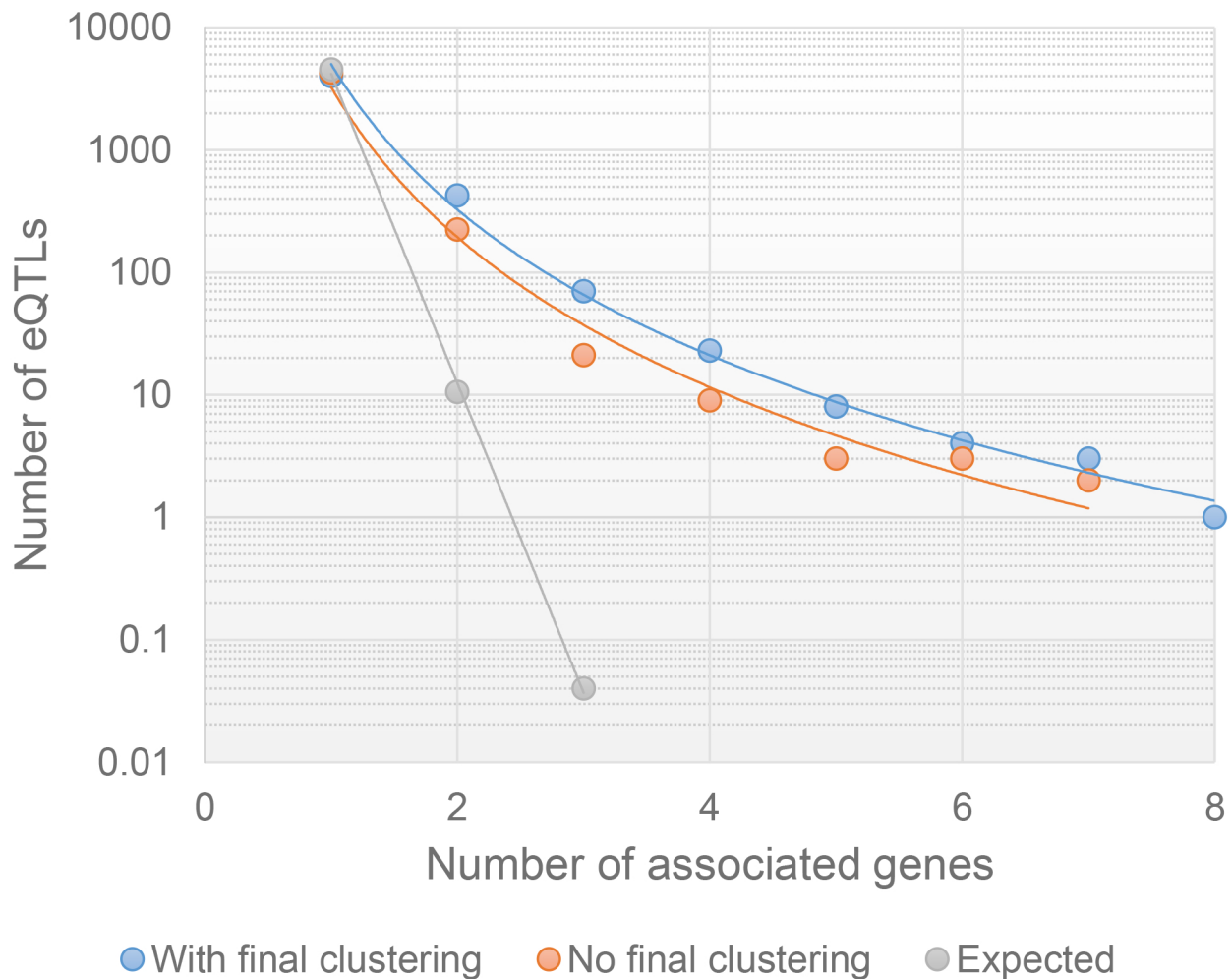

Supplement: S8 Fig — The observed number without the subsequent grouping step is shown i.e. without grouping eQTLs across genes that were redundant. (PDF) [file pgen.1006673.s008.pdf]
